# Understanding Addiction Using Animal Models

**DOI:** 10.3389/fnbeh.2019.00262

**Published:** 2019-11-29

**Authors:** Brittany N. Kuhn, Peter W. Kalivas, Ana-Clara Bobadilla

**Affiliations:** Department of Neuroscience, Medical University of South Carolina, Charleston, SC, United States

**Keywords:** addiction models, preclinical studies, DSM-V, drug seeking behavior, relapse activity

## Abstract

Drug addiction is a neuropsychiatric disorder with grave personal consequences that has an extraordinary global economic impact. Despite decades of research, the options available to treat addiction are often ineffective because our rudimentary understanding of drug-induced pathology in brain circuits and synaptic physiology inhibits the rational design of successful therapies. This understanding will arise first from animal models of addiction where experimentation at the level of circuits and molecular biology is possible. We will review the most common preclinical models of addictive behavior and discuss the advantages and disadvantages of each. This includes non-contingent models in which animals are passively exposed to rewarding substances, as well as widely used contingent models such as drug self-administration and relapse. For the latter, we elaborate on the different ways of mimicking craving and relapse, which include using acute stress, drug administration or exposure to cues and contexts previously paired with drug self-administration. We further describe paradigms where drug-taking is challenged by alternative rewards, such as appetitive foods or social interaction. In an attempt to better model the individual vulnerability to drug abuse that characterizes human addiction, the field has also established preclinical paradigms in which drug-induced behaviors are ranked by various criteria of drug use in the presence of negative consequences. Separation of more vulnerable animals according to these criteria, along with other innate predispositions including goal- or sign-tracking, sensation-seeking behavior or impulsivity, has established individual genetic susceptibilities to developing drug addiction and relapse vulnerability. We further examine current models of behavioral addictions such as gambling, a disorder included in the DSM-5, and exercise, mentioned in the DSM-5 but not included yet due to insufficient peer-reviewed evidence. Finally, after reviewing the face validity of the aforementioned models, we consider the most common standardized tests used by pharmaceutical companies to assess the addictive potential of a drug during clinical trials.

## Introduction

“*All models are wrong, some models are useful*.”

George Box

The main difficulty with modeling drug addiction using nonhuman animals is capturing an inherently complex behavioral pathology using relatively simple behavioral protocols (Spanagel, 2017; Müller, 2018). Environmental circumstances, behavioral traits and genetic factors all interplay with one another and affect an individual’s susceptibility to acquiring and maintaining the use of an addictive substance, as well as relapse propensity (Everitt and Robbins, 2016). Regardless, creating better preclinical models of drug addiction is essential for elucidating the neurobiological mechanisms that contribute to addiction-related behaviors, and creating better treatment options for those afflicted with addiction. In this review article, we build on the existing literature (García Pardo et al., 2017; Lynch, 2018) and discuss the various models that exist for studying addiction-related behaviors in animals, including individual variation in addiction-related behaviors, and commonalities between drug addiction and certain behavioral addictions. We then focus on how these models are used to assess abuse potential by pharmaceutical companies.

In models such as behavioral sensitization and conditioned place preference (CPP), an animal is non-contingently administered a drug, allowing drug delivery independent from the motivation to take the drug to be assessed. More commonly used are drug self-administration (SA) models, whereby drug delivery is contingent upon the animal’s motivation to take the drug. SA models are constantly evolving, with the emphasis being placed on the importance of the duration of drug experience (i.e., session length) and the temporal pattern of drug delivery as a means to better model the transition to addiction. The motivation for drug-taking can also be assessed using multisymptomatic training paradigms and behavioral economics. Factors that contribute to reinstated drug-seeking behavior, including environmental contexts, cues, stressors and the drug itself are briefly discussed, along with procedures where drugs are challenged by alternative rewards.

Independent of modeling addiction-related behaviors, we emphasize the importance and advantages of modeling individual variation within these behaviors, as is seen in humans. For example, measures of impulsivity have been shown to predict addiction liability. We also discuss two well-established models that capture individual variation in addiction-related behaviors: the high-responder/low-responder model, and the sign-tracker/goal-tracker model. While the high-responder/low-responder model captures individual variation in the acquisition of drug-taking behavior, the sign-tracker/goal-tracker model captures individual variation in relapse propensity. Thus, the two models independently capture two different phases of addiction.

Next, we discuss behavioral addictions that share several similarities with drug addiction, with an emphasis on gambling disorder. There is a high prevalence rate between those diagnosed with gambling disorder and those with a substance abuse disorder (17% for illicit drugs, 28% for alcohol dependence according to Lorains et al., 2011) and not surprisingly the criteria to diagnose the two are very similar (for review see Rash et al., 2016). As with drug addiction, accurately modeling behaviors associated with gambling disorder using rodents are complex. The growing prevalence and efforts to assess and diagnose exercise addiction are also briefly discussed.

We end this review with a discussion on the models used by pharmaceutical companies to assess the abuse potential of possible medications for the treatment of addiction. It is for this reason that critical evaluation of models used to assess addiction-related behaviors is particularly necessary, as these models are being used to evaluate treatment efficacy.

## Non-Contingent Models: Behavioral Sensitization, Conditioned Place Preference and Runway Model

Models based on non-contingent (i.e., experimenter-administered) drug exposure are simple and quick to set up. Because of these advantages, many studies have used them to identify key reward-related neurobiological substrates and how drug exposure alters them. See Table 1 for a succinct summary of advantages and limitations of these models.

**Table 1:** Brief summary of preclinical behavioral models most currently used to study the neurobiology of addiction: non-contingent drug administration and contingent drug self-administration (SA).

Model	Advantages	Limits
**Non-contingent drug administration**		
Behavioral sensitization	Long-lasting Shared by all drugs Cross-sensitization amongst drugs	Lack of animal-driven behavior Stereotypies at high doses Not exclusive to drugs of abuse Poor face validity
Conditioned Place Preference (CPP)	Drug-free testing Establishes rewarding or aversive properties	Lack of animal-driven behavior Not exclusive to drugs of abuse
Runway Model	Can be adapted to contingent drug delivery Drug-free testing Establishes rewarding or aversive properties	Requires initiation training with other rewards Not exclusive to drugs of abuse
**Contingent—drug self-administration (SA)**		
Short Access (ShA)	Short training sessions Reliably shows escalation of intake and relapse behavior	Does not capture compulsive drug-taking behavior
Long Access (LgA)	Greater escalation of intake, higher break-points and greater drug-induced reinstatement compared to ShA	Long training sessions
Intermittent Access (IntA)	Captures temporal pattern of drug intake observed in humans Greater motivation to work for the drug and cue-induced reinstatement compared to ShA and LgA	More complex behavioral training compared to ShA and LgA
2-Bottle choice-Ethanol	Simple set up, no invasive surgery Good correlation between intake and blood concentration Good face validity	Low consumption Initiation training with other rewards
Drink in the Dark (DID)-Ethanol	No confounding with other reward Good face validity for binge-drinking	Strain-specific
Chronic Intermittent Ethanol (CIE)	Good face validity (increased voluntary consumption)	Long protocol

### Behavioral Sensitization

The behavioral sensitization model is at the heart of the incentive-sensitization theory of addiction developed by Robinson and Berridge (1993). The model is based on the potentiation of drug-induced locomotion after repeated non-contingent exposure to a constant drug dose. Behavioral sensitization is usually divided into two phases: the induction (or initiation) and the expression phase. During the induction phase, it is possible to measure the molecular and cellular modifications directly induced by drug exposure. The expression, tested with a drug challenge and after a variable withdrawal, is generally attributed to the long-term effects of the aforementioned drug-induced changes. Behavioral sensitization requires D1-dopaminergic receptor activation in the ventral tegmental area (VTA; Vezina, 1996) and AMPA-mediated glutamatergic transmission in the nucleus accumbens (NAc), the latter being shared with most models of drug seeking (Bell and Kalivas, 1996; Pierce et al., 1996; Famous et al., 2008). Sensitization to all drugs of abuse has also been shown to correlate with a sustained hyper-reactivity of noradrenergic and serotonergic systems in the locus coeruleus and dorsal raphe, respectively (Tassin, 2008; Doucet et al., 2013).

Behavioral sensitization has many advantages. First, drug delivery is simple, as it relies on experimenter-administered intraperitoneal injections. Depending on the experimental timeline and withdrawal periods, sensitization can be rapidly induced since potentiation can be measured after only a few drug injections, or even a single injection (Magos, 1969; Robinson et al., 1982; Vanderschuren et al., 1999a; Valjent et al., 2010). However, it is possible to use sensitization to study the long-term effects of chronic drug-exposure, one rat study showing amphetamine sensitization lasting up to a year (Paulson et al., 1991). But beyond these technical strengths, an important characteristic of behavioral sensitization is that most drugs of abuse, including cocaine (Post and Rose, 1976), amphetamine (Segal and Mandell, 1974), morphine (Babbini and Davis, 1972), ethanol [in certain mouse strains only, never shown in rats (Didone et al., 2008; Bahi and Dreyer, 2012a,b)], and nicotine (Clarke and Kumar, 1983), induce sensitization in rodents. Notably, no behavioral sensitization has been shown with Δ^9^-tetrahydrocannabinol (THC; Varvel et al., 2007). Throughout the drug spectrum, sensitization remains sensitive to individual vulnerabilities to stress and genetics factors, as demonstrated by the wide range of behaviors observed with different rodent strains (Phillips et al., 1997).

Most importantly, drugs often cross-sensitize, which means that potentiated response to one drug is observed after induction with another drug. Cross-sensitization has indeed been observed between psychostimulants such as amphetamine and cocaine (Bonate et al., 1997; Vanderschuren et al., 1999a), but also across different drug classes like amphetamine and morphine (Vezina and Stewart, 1990; Vanderschuren et al., 1997), with the exception of one study in rats where cross-sensitization was lacking (Vanderschuren et al., 1999b). Surprisingly, although THC does not induce behavioral sensitization as mentioned earlier (Varvel et al., 2007), it cross-sensitizes with opioids (Lamarque et al., 2001; Cadoni et al., 2008) and amphetamine (Lamarque et al., 2001) in rats. However, cannabinoid agonist HU 210 failed to cross-sensitize with morphine or alcohol (Hagues et al., 2008). Interestingly, pre-exposing animals to drugs, thus inducing sensitization, potentiates CPP (Lett, 1989; Gaiardi et al., 1991; Shippenberg and Heidbreder, 1995; Shippenberg et al., 1996; Meririnne et al., 2001; Harris and Aston-Jones, 2003a,b) and SA behaviors (Horger et al., 1990, 1992; Piazza et al., 1990; Valadez and Schenk, 1994; Pierre and Vezina, 1998; Covington and Miczek, 2001) compared to non-sensitized controls. These results support the idea that chronic non-contingent drug exposure used in the sensitization model allows studying the neurobiological pathways shared by all drugs.

Despite all the advantages of this model, behavioral sensitization faces major downsides that contributed to the field increasingly shifting towards other models. Before all else, the face validity of this model is limited as sensitization in humans is challenging to demonstrate, although some studies measured a potentiation of certain symptoms such as activity and energy levels, mood or speech quantity after repeated amphetamine challenges that could be compared to behavioral sensitization (Strakowski et al., 1996; Strakowski and Sax, 1998; Boileau et al., 2006). Chronic amphetamine use has also been linked to progressive augmentation in paranoid behaviors (Kalivas and Stewart, 1991). Another characteristic of this model contrasting with the clinic resides in the fact that only a few drug injections can induce sensitization in rodents, whereas in humans a large number of exposures over time often precede abuse. Because of this, it has been argued that this model can be useful to better understand the initial phases of drug intake that influence, but does not provide a complete picture, of the transition to substance use disorder (Vanderschuren and Pierce, 2010). Finally, cross-sensitization is not limited to drugs, but also extends to stressors such as foot shock (Herman et al., 1984; Sorg, 1992), restrain stress (Robinson et al., 1985) or tail pinch (Antelman et al., 1980). In addition to stress, amphetamine-sensitized animals show facilitation of sexual behavior (Fiorino and Phillips, 1999a,b), whereas rats sensitized to morphine display increased interest for food, as well as sexual and social behaviors (Nocjar and Panksepp, 2007). These results suggest that drug-sensitization induces an unspecific activation of the reward pathways, which contradicts Diagnostic and Statistical Manual (DSM) criteria of addicted individual’s disinterest for natural, social or professional rewards (APA, 2013).

### Conditioned Place Preference (CPP)

CPP (also known as place preference conditioning, or PPC) allows testing the rewarding or aversive properties of an experience or a stimulus. A multitude of variations of the CPP model exist, yet the standard use remains to associate an experience, such as non-contingent drug delivery, to a recognizable context, often a chamber with definitive cues identifiable by the animal. In parallel, animals are also exposed to a different neutral context. After a first phase of pre-conditioning to assert no innate preference of the animal for one of the contexts, the acquisition phase consists of pairing the drug to one context. Pairing can be achieved after single or repeated exposures, depending on the drug doses or timing exposures. On test day, or post-conditioning phase, the animal is allowed to explore the contexts freely. When the time spent in the paired context is longer to the time spent in the unpaired one, the stimulus is concluded to be rewarding. The opposite result grants aversive effects to the stimulus, defined as Conditioned Place Aversion (CPA). CPP was first shown with morphine (Beach, 1957), and a multitude of studies later showed that all drugs of abuse, including cocaine, amphetamine, methamphetamine, nicotine, alcohol and cannabis also induced CPP (Bardo and Bevins, 2000; García Pardo et al., 2017). Along with SA, CPP is the most commonly used model to test abuse potential of a new drug during clinical trials (see later section). The popularity and abundant use of this model relies on its simplicity and promptness. Moreover, the drug of interest is typically not administered on testing day, thus allowing the determination of its rewarding properties and measurement of enduring neurobiological changes induced by the drug without causing massive neurotransmitter release. It also provides a tool to definitively establish aversive effects through avoidance, which lacks in the self-administering paradigm (i.e., lack of responding to a drug can be interpreted as the drug having aversive or neutral properties). Importantly, individual differences and susceptibilities to the rewarding effects of drugs can be measured using this paradigm. While reviewing how the novelty-seeking endophenotype impacts psychostimulant responses, Arenas et al. (2016) summarized the potentiated responses of high responding rats to sub-threshold doses of amphetamine and cocaine compared to low responding animals (see the “Individual Variation in Addiction-Related Behaviors” section below for more detailed definition of the High-responder/Low-responder model). This potentiated response could be linked to the corticotropin-releasing factor (CRF) since a study found that mice continually overexpressing CRF show potentiated cocaine CPP only in mice displaying low behavioral reactivity to novelty in contrast to mice with high locomotor response to novelty (Kasahara et al., 2015). More recently, a study established a positive correlation between rats exhibiting risk-taking behaviors and methamphetamine-induced CPP (Takahashi et al., 2019), corroborating the ability to detect individual differences using this paradigm. However, CPP is not specific to abused substances, since natural rewards such as food (Cason et al., 2010), novelty (Klebaur and Bardo, 1999), physical exercise (wheel running; Antoniadis et al., 2000) and sexual behavior (Paredes, 2009) induce robust CPP. Similar to the behavioral sensitization and SA models, CPP heavily relies on motor capacities, thus requiring appropriate controls to assess possible sedative or anxiolytic effects of the drug that are not present when testing the animal in a drug-free state.

Using receptor-specific agonists and antagonists, many studies (for an extensive review see Tzschentke, 2007) have established a necessary role of the usual suspects within reward circuitry (dopaminergic, glutamatergic, GABergic, cholinergic, noradrenergic and serotonergic systems) in the induction and maintenance of CPP. Consistent with this idea of shared reward-related mechanisms, inducing CPP often potentiates the behavior observed in other models of addiction as seen in a study where expression of cocaine behavioral sensitization is only observed in the compartment paired with the drug during CPP, and not in a novel compartment (Duvauchelle et al., 2000). Using CPP and CPA can also help elucidate heterogeneous behaviors in other models. Supporting this, the authors of a study comparing animals self-administering cocaine at a high and stable level to animals failing to do so elegantly show that self-administering animals exhibit cocaine CPP, while non-self-administering ones show CPA (Rademacher et al., 2000). These results argue that cocaine effects are appetitive for some animals but aversive for others. However, inducing CPP with one reward does not always predict a potentiated reward-induced behavior in other models, as reported in a study that found no correlation between the magnitude of novelty-induced CPP and the degree of amphetamine SA (Klebaur et al., 2001). Another study reported that ethanol consumption during the SA pre-exposure phase negatively correlates with ethanol-induced CPP in mice (Nocjar et al., 1999).

In some studies, CPP is also used to model relapse and dissect the neurobiology of drug-seeking (for extensive review, see Aguilar et al., 2009). Prior to CPP reinstatement, animals undergo extinction training, consisting in either exposing the animal to the previously reward-paired context without administering the reward or administering sham injections in the paired and unpaired contexts (Epstein et al., 2006; Aguilar et al., 2009). CPP reinstatement, shown for cocaine, amphetamine, methamphetamine, morphine, heroin, nicotine, ethanol and MDMA, can be induced by a priming dose of drug or different types of stress, including footshock, immobilization and forced swim (Liu et al., 2008; Aguilar et al., 2009). A study further demonstrated robust cocaine CPP reinstatement induced by conditioned fear stimuli, in this case an odor or a tone previously paired with footshock (Sanchez and Sorg, 2001). In a study testing the establishment, maintenance, extinction and reinstatement of cocaine CPP, authors showed that once developed, place preference endures for several weeks and is rapidly reinstated after extinction training following cocaine priming injections (Mueller and Stewart, 2000). Similar to the induction and maintenance part of CPP, several neurotransmitters have been shown to drive reinstatement, including glutamatergic, dopaminergic and noradrenergic transmissions. The role of these systems seems to be highly dependent on reinstatement modality, i.e., drug priming or stress (Aguilar et al., 2009).

### The Runway Model

The runway model has been used to evaluate opponent aversive and rewarding components of drugs. It was developed to study goal-directed behaviors with natural rewards (Hull, 1934; Crespi, 1942; Miller, 1944). From the start compartment, animals learn to cross a 6-foot long straight corridor (the runway) to reach the goal compartment, where the reward is delivered (Ettenberg, 2004, 2009). Prior to drug exposure, food-deprived animals are trained to enter the goal compartment for food pellets. Subsequently, entering the goal compartment is associated with drug delivery. The run time, i.e., the time the trained animal takes to reach the goal compartment, is the resultant of approach and avoidance behaviors and is interpreted as an index of motivation/aversion for the reward. While in some cases the drug-seeking is delivered in a noncontingent fashion, the model has been adapted to more recent techniques such as i.v. drug self-administration (Geist and Ettenberg, 1990) or optogenetics (Jhou et al., 2013). The runway model allows characterization of the ambivalent properties of drugs, which have been shown with most drugs including cocaine, amphetamine, heroin, morphine, MDMA and ethanol (for review see Ettenberg, 2009).

## Contingent Models of Addiction-Related Behaviors

The models presented here rely on operant learning during repeated exposure to the drug of interest. During SA sessions an animal performs an action (e.g., lever deflection or nose port entry) in order to receive an infusion of the drug. There is consensus in the addiction field that initial exposures to the drug mainly impact the prefrontal cortex (PFC), driving goal-directed behavior, and the mesolimbic regions, including the NAc, are key regions in the integration of reinforcing stimuli (Hopf and Lesscher, 2014). As the training develops, the dorsal striatum has been shown to take a major role in maintaining drug intake (Belin and Everitt, 2008). Below we discuss the main SA paradigms currently used in drug addiction research, followed by a discussion on the various ways to assess the motivation for taking a drug and relapse propensity. See Table 1 for a brief summary of advantages and limitations of drug self-administration models and Table 2 for models of motivation and drug-seeking and relapse models mentioned below.

**Table 2:** Brief summary of preclinical behavioral models most currently used to study the neurobiology of addiction: motivation and seeking in contingent models, models including alternative rewards.

Model	Advantages	Limits
**Contingent—motivation for drug taking**		
Progressive Ratio (PR)	Simple task Can be done several times throughout training	Direct comparisons between different reinforcers not possible Reward on-board during test
Behavioral Economics	Can measure several parameters of motivation within one session Can be done several times throughout training Direct comparisons between different reinforcers possible	Complex data analysis
DSM Related	Modeled directly from DSM criteria Good face validity	Requires the use of several different behavioral paradigms Long protocol
**Contingent—drug seeking and relapse**		
Cued-Reinstatement	Two types: discrete and discriminative cues Simple set-up Good face validity	Action precedes the reward-cue in discrete cue-induced reinstatement (usually reverse in human population)
Context-Reinstatement	Can assess the effects of a compilation of cues on different sensory modalities on drug-seeking behavior	Complex SA procedures (i.e. multiple contexts needed)
Prime-Reinstatement	Simple set-up Good face validity	Route of administration normally different than during SA\item Reward on-board during test
Stress-Reinstatement	Environmental and interoceptive stressors can be used Shown to increase relapse across several classes of drugs	Complex training with certain stressors Difficult to model complex human psychological stressors
**Contingent drug seeking in presence of alternative rewards**		
Any model presenting competing choice between drugs and other rewards	Good face validity: models closer to human experience (not-limited to drug exposure exclusively) Myriad of alternative rewards available (appetitive foods, social interactions, enriched environment, etc.)	Complex study of the neurobiology of each reward Might require longer training and protocols Might require larger n and additional controls

### Drug Self-administration Models

Animal drug SA paradigms have significantly evolved since the inception of the technique in 1962 (Weeks, 1962), as more attempts are being put forth to more accurately model drug-taking behavior in humans. A commonly used paradigm, short-access (ShA) training, involves SA sessions that generally last between 1 and 3 h. ShA paradigms reliably show an increase in drug-taking behavior and reinstatement of drug-seeking behavior, two features of human addiction. However, despite its proliferous use, arguments have been made that ShA sessions capture drug-taking behavior, but not behavior that is representative of the transition to addiction (Ahmed and Koob, 1998). That is to say, ShA may only address recreational drug use, and not the escalation to compulsive drug taking that is seen in human addicts (for review see Roberts et al., 2007). To address this limitation, a long-access (LgA; Spanagel et al., 1996) training procedure was developed that consists of SA training sessions lasting a minimum of 6 h (Ahmed and Koob, 1998), with some lasting as long as 12 h (Lucantonio et al., 2015; Cocker et al., 2019). Compared to ShA sessions, rats undergoing LgA training show greater escalation in drug-taking behavior (Ahmed and Koob, 1998; Mantsch et al., 2004; Mandt et al., 2015), are more motivated to work for an infusion of cocaine (Paterson and Markou, 2003; Hao et al., 2010), and show greater cocaine-primed drug-seeking behavior (Mantsch et al., 2004; Knackstedt and Kalivas, 2007). The neurobiological mechanisms mediating behavior as a result of these two training paradigms also appear to differ, specifically in respect to neuroplasticity within the striatum (Purgianto et al., 2013; Ducret et al., 2016).

Rats have continuous access to a drug during LgA and ShA training, however, the temporal pattern of drug delivery has been argued to also play a critical role in the transition to addiction. Human addicts have been reported to take cocaine intermittently whereby a large quantity of cocaine is consumed within a short time span, followed by a period of no drug use before consuming cocaine again (Allain et al., 2015). This intermittent pattern of drug-taking is believed to cause a constant spiking of brain-cocaine concentration levels and contribute to addiction-related behaviors (Zimmer et al., 2011). The intermittent access (IntA) training procedure emulates this behavior in a rodent model. During this task, rats are allowed to self-administer during 5 min bins that are separated by 25 min periods where the drug is not available (Zimmer et al., 2011). This procedure results in a high level of consumption during drug-available periods (Allain et al., 2018) and fluctuations in brain-cocaine concentration levels (Zimmer et al., 2012). While rats trained to self-administer cocaine using LgA procedures consume more drug, rats trained using IntA show greater motivation to work for cocaine compared to rats in LgA or ShA training (Zimmer et al., 2012). Rats also show greater cue-induced drug-seeking behavior following IntA training compared to LgA or ShA (Kawa et al., 2016, 2019). IntA results in greater dopamine concentrations within the NAc core following a single infusion of cocaine compared to LgA training, and dopamine levels correlated with several measures of motivation for cocaine (Kawa et al., 2019). IntA training, but not LgA or ShA, using psychostimulants also results in sensitization of NAc dopamine transports (Calipari et al., 2013, 2014). Taken together, it is apparent that while ShA, LgA and IntA all produce drug-taking and drug-seeking behavior, the behavioral paradigms differ in several other measures of motivation for a drug and resultant neurobiological effects, both of which are factors that should be taken into consideration during experimental design.

## Motivation for Drug-Taking Behavior

Motivation for a drug can be measured independent of the quantity of drug consumed or pattern of intake. Progressive ratio tests and behavioral economics can be used to assess the reinforcing properties of a reward as the price and/or demand for the drug is manipulated. Adapting DSM criteria on substance use disorders to behavioral tests are also used to more directly translate data from rodent models to human addiction.

### Progressive Ratio

Progressive ratio schedules are within-session procedures where the cost of a reward exponentially increases with each subsequent trial (Hodos, 1961; Roberts and Richardson, 1992; Richardson and Roberts, 1996). Using this paradigm, the motivation of the animal to work for a reward can be measured, with the maximum number of responses an animal makes in order to receive the reward referred to as the “break-point” (BP). BPs can be taken at several time points in an experiment, yielding insight into how the reinforcing properties of a drug change over the course of drug SA training. Behavior during this test is particularly sensitive to drug dose (Roberts et al., 1989), injection speed (Woolverton and Wang, 2004; Liu et al., 2005), and availability of drug during SA as well as length of forced abstinence (Morgan et al., 2002, 2005). Due to these factors, comparing BPs for the same reward between studies is often difficult. Furthermore, BPs are not comparable between different reinforcers, as they are not standardized to a baseline threshold, as is common using demand curves (discussed below). Nevertheless, a progressive ratio test is a useful tool to assess the motivation of an animal to work for a drug, and thus track the transition to addiction in animal models (for review see Roberts et al., 2007).

### Behavioral Economics: Demand Curve Analysis

Behavioral economics approaches, specifically demand curve analyses, have become more widely used due to their unique ability to measure several parameters of motivation for a drug during SA (Bickel et al., 1993, 2011; Hursh and Winger, 1995). A demand curve is the effort an individual is willing to expend for a reward at various prices (Hursh, 1980), and so the cost or price of a reward is a function of that effort (Hursh et al., 1988). Demand curves can be generated within a single SA session using a threshold procedure (Oleson and Roberts, 2009; Oleson et al., 2011; Bentzley et al., 2013). These sessions typically last 110 min, and every 10 min the dose of drug available decreases according to a quarter logarithmic scale. At the conclusion of the session, a demand curve is fit to the data, and several variables are generated that yield insight into the reinforcing properties of the reward (Hursh and Silberberg, 2008). Recent models of demand curve analysis have used a focused fitting approach, whereby data points that are generated when brain-cocaine concentrations greatly fluctuate, generally at the start of the session when the animal is “loading” on the drug or toward the end of the session when the price is beyond what the animal is willing to work for the drug, are removed from analysis (Bentzley et al., 2013). This has been shown to result in a demand curve that more accurately represents the behavior of the subject (Bentzley et al., 2013).

The following variables are calculated from the demand curve: Q_o_, P_max_, O_max_ and α. Q_0_ is a measure of the “hedonic set point” (Ahmed and Koob, 1998, 1999), or the drug intake when the effort to acquire the drug is low. It thus acts as a general measure of consummatory behavior (Oleson et al., 2011). Because the price of the reward is low, Q_o_ is a function of demand only. In contrast, P_max_ is a function of elasticity. Elasticity refers to the rate at which the slope of the demand curve changes as the price for the reward increases (Hursh, 1980). A demand curve showing more elasticity is indicative of an individual showing less effort to consume the reward as the price increases. P_max_ is the maximum price (responses/mg reward) an individual will pay to consume the reward, or rather the maximum effort the animal will expel to maintain its hedonic set point (i.e., Q_o_; Hursh, 1991). It is not too surprising then that P_max_ values have been shown to correlate with the BP in a progressive ratio test (Rodefer and Carroll, 1997; Bickel and Madden, 1999; Lenoir and Ahmed, 2008; Oleson and Roberts, 2009). The O_max_ value, or the maximum number of responses made at P_max_, is a function of both demand and elasticity (Hursh and Winger, 1995). This value is unique in that it is the only variable generated from a demand curve that reliably predicts the success of drug addiction treatment (MacKillop and Murphy, 2007). The last variable, α, is known as the “essential value” of a reward and is the slope of the demand curve (Hursh and Silberberg, 2008). The motivation to continue to work for the drug as price increases is inversely related to α, such that rewards with a higher essential value have smaller α values, and show less elasticity (Bentzley et al., 2013). Alpha can also be tracked across several time periods throughout SA training to see how the essential value of the drug changes with increased drug experience (Christensen et al., 2008b). An advantage of α in comparison to the other variables is that it is inherently normalized to Q_o_, allowing for α values to be directly compared between reinforcers. In fact, food as a reward has a greater essential value compared to both cocaine (Christensen et al., 2008a) and methamphetamine (Galuska et al., 2011). Though P_max_ and O_max_ are not normalized to Q_o_, they can manually be such that these values are also able to be compared across different rewards (Ko et al., 2002; Winger et al., 2006; Wade-Galuska et al., 2007). Overall, though data analysis is complex, demand curve analysis affords researchers the ability to parse the multiple components of motivation for drug-taking behavior within a single session and allows for the direct comparison of these components between different rewards.

### Modeling DSM-Related Drug Addiction Behaviors

Another consideration when modeling addiction-related behaviors in animal models is incorporating the diagnostic criteria within the DSM for substance use disorders in humans. To that end, efforts have been put forth to model some of the criteria in animal models in order to create better preclinical models of drug addiction (Deroche-Gamonet et al., 2004; for review see Belin-Rauscent et al., 2016). Criterion often modeled includes: compulsive drug-seeking behavior when the drug is not available, high levels of motivation for the drug, and continuing to take drug despite the co-occurrence of adverse consequences (Deroche-Gamonet et al., 2004). In a rodent model, these criteria are applied by measuring drug-seeking behavior during periods of signaled no drug availability, using progressive ratio tests, and pairing a foot shock with reward consumption (for review see Belin-Rauscent et al., 2016). By using this multi-symptomatic model, rats can be separated based on the number of criteria met for a substance use disorder diagnosis, and interestingly the percent of rats that meet all criteria is very similar to the percent of human drug addicts that meet DSM criteria, further strengthening the validity of using this model (Deroche-Gamonet et al., 2004). Another advantage of this model is the ability to assess individual differences in behavioral traits and neurobiological factors that may contribute to an addicted phenotype (Belin et al., 2008, 2009, 2011; Kasanetz et al., 2010; Kawa et al., 2016).

## Models of Reinstatement of Drug-Seeking Behavior

The biggest obstacle in the treatment of drug addiction is the high rates of relapse following exposure to environmental stimuli (e.g., cues, contexts, stressors) associated with prior drug-taking behavior (Shaham et al., 2003; Bossert et al., 2013). The most common methods for examining relapse behavior in animal models is *via* tests for cue-induced [both discrete (Meil and See, 1996) and discriminative (Weiss et al., 2000)], context-induced (Crombag and Shaham, 2002), drug-primed (de Wit and Stewart, 1981) and stress-induced (Shaham and Stewart, 1995) reinstatement. Tests for reinstatement generally occur following a period of abstinence, such as forced abstinence, voluntary abstinence, extinction training, or a combination of these procedures. While each reinstatement test isolates a specific factor that contributes to drug-seeking behavior, in humans, several of these factors likely co-occur and result in relapse. However, by studying each of these models separately, we are able to assess similarities and differences in the neurobiological mechanisms that mediate each mode of relapse (for review see Crombag et al., 2008; Bossert et al., 2013).

Cues associated with the drug-taking experience can result in craving (Childress et al., 1988, 1993) and ultimately drug relapse (see Bossert et al., 2013). There are two common types of cues used during SA training that differ based on their contingency of presentation: a discrete cue and a discriminative stimulus (DS). A discrete cue is one that is localizable and directly tied to operant responding for drug delivery (e.g., light above the lever), thus presentation of a discrete cue is contingent upon drug-taking behavior. During discrete cue-induced reinstatement, the action during SA that resulted in drug delivery and presentation of the discrete drug-associated cue now only results in the presentation of the cue. Therefore, during these tests, the conditioned reinforcing property, or the ability of the cue to invigorate ongoing behavior, of the reward-paired cue is being assessed. In humans, however, though presentation of the reward-cue invigorates drug-seeking behavior, oftentimes drug-seeking behavior precedes presentation of a reward-cue. While using a single discrete cue can evoke drug-seeking behavior, using a compound discrete stimulus, such as a cue-light and tone pairing, results in a more robust reinstatement (Kruzich et al., 2001). In contrast to a discrete cue, a DS signals when the reward is, or is not, available during SA training sessions. That is, a DS operates as an “occasion setter” (Crombag et al., 2008), signaling when operant responding for the drug will (positive DS), or will not be (negative DS), reinforced (e.g., house light turning on and off). Thus, presentation of the DS is not contingent upon drug-taking behavior. During discriminative cue-induced reinstatement tests, the positive or negative DS is noncontingently presented, and the ability of the DS to affect subsequent drug-seeking behavior is analyzed. While the use of a DS is oftentimes classified as a type of cue-induced reinstatement, some have argued that it shares more properties with context-induced reinstatement (Weiss, 2005; Trask et al., 2017).

Like cues, exposure to contexts associated with the drug-taking experience can result in relapse in humans (Wikler, 1973; O’Brien et al., 1992). A context is a compilation of several cues where no single cue predicts drug availability more than another. To test the ability of a context to invigorate drug-seeking behavior, animals are trained to self-administer drugs in one context (Context A), extinguished in a separate context (Context B), and then reintroduced to Context A for the reinstatement test (Bouton and Bolles, 1979; Crombag and Shaham, 2002; Fuchs et al., 2005). In addition to environmental cues and contexts evoking drug-seeking behavior, interoceptive cues associated with the drug experience following abstinence can also result in craving (Jaffe et al., 1989) and elevated intake (de Wit and Chutuape, 1993). Tests for drug-primed reinstatement involve the non-contingent delivery of the drug prior to being tested in extinction conditions, thus the reinstating properties of a drug are being tested while the drug is on board. In contrast to intravenous delivery as during SA, the drug prime is usually administered subcutaneously or intraperitoneally, leading to possible pharmacokinetic differences in the drug’s effect. Regardless, prime reinstatement remains the only test that isolates the ability of the drug itself to invigorate drug-seeking behavior.

The ability of different environmental and interoceptive stressors to invigorate drug-seeking behavior has also been explored. During these tests, a stressor is generally administered to the animal *prior* to the session starting. The main stressors used are food deprivation (Shalev et al., 2000), intermittent foot-shock (Shaham and Stewart, 1995), and administration of yohimbine (Shepard et al., 2004), a drug that causes anxiety- and stress-like effects. While it is difficult to model the complex psychological and physical stressors relevant to the human condition using animal models, the aforementioned stressors have been shown to potentiate drug-seeking behavior across several classes of drugs (for review see Mantsch et al., 2016).

There are several similarities and differences between the neurobiological mechanisms that mediate drug-seeking behavior using the different models of reinstatement. However, such detail is not within the scope of the current review, but several reviews exist that proficiently address the neurobiology associated with different animal relapse models (see Crombag et al., 2008; Bossert et al., 2013; Mantsch et al., 2016).

## Alternative Rewards

Most operant models currently used to dissect the neurobiology underlying abuse disorders present very restricted options to animals; they can choose between self-administering the reward or not. However, a growing number of studies prove that when given broader choices, the vast majority of animals recoil from drug rewards.

Early in the development of the addiction field, a few rare studies measured the desire of dogs or chimpanzees pre-treated with drugs to choose cocaine or morphine over food (Tatum and Seevers, 1929; Spragg, 1940), in an attempt to model addiction-like phenotypes in animals. More recently, Lenoir et al. (2007) published an elegant study that surprised many: when given the mutually exclusive option between saccharin-sweetened water or cocaine, 94% of the animals preferred the sweet water over intravenous cocaine. Rats established this preference after multiple cocaines and sweet water samplings, consistently over 15 days of training. Importantly, when cocaine was present, maximal lever sampling and locomotor sensitization confirmed its rewarding and locomotor effects. Based on these results and subsequent work, Ahmed et al. (2013) argued that the field might be limited by using models lacking competing choices to study addiction, a disorder altering value-based decision-making. To study craving after experiencing several rewards, one study tested reinstating animals after they underwent food and cocaine SA, followed by choice tests and extinction training (Tunstall and Kearns, 2014). While the majority of rats chose to self-administer food, cocaine-primed reinstatement induced a significant increase in lever pressing of the cocaine-associated lever. Footshock and food-primed induced reinstatements however only induced a mild, non-specific increase of responding in both levers. The authors conclude that cocaine seeking can prevail over food seeking when cocaine is on board during primed reinstatement. A follow-up study established that when choosing between cocaine or grain pellets, rats still preferred pellets. However, cue-induced reinstatement following extinction training showed cocaine craving, as measured by a significant increase in lever pressing for the previously cocaine-associated lever (Tunstall and Kearns, 2016). When choosing between grain and sucrose pellets, the majority of rats self-administered sucrose over grain pellets and also responded more to the sucrose-associated lever during cued-reinstatement. The cocaine/sucrose paradigm was not tested. These results argue for a strengthening of the cocaine-associated cue despite cocaine not being the preferred option during SA.

Recently, Venniro et al. (2018) elegantly developed an operant model of choice between drugs and social interaction and showed that operant social reward prevented methamphetamine and heroin SA, even in rats exhibiting a high addiction score (Deroche-Gamonet et al., 2004). It also prevented methamphetamine incubation of craving and relapse, through protein kinase C-δ -expressing neurons in the central amygdala and inhibition of activity in the anterior ventral insular cortex (Venniro et al., 2018). These results are consistent with what is observed in humans, where greater social support and integration predicted lower risk of relapse for alcohol, opiates and cigarette smoking (Havassy et al., 1991). Another innovative study showed how social interactions profoundly affect decision-making and firing of dopaminergic cells in the VTA by analyzing the behavior of mice living in Souris City, a large environment shared by a large community of peers (Torquet et al., 2018). Based on measurements obtained after experimenter-induced social re-organizations, the authors highlight the importance of social environments on animals’ individual profiles and goal-directed decision-making.

The effects of alternative rewards are not only observed in contingent models. Solinas et al. (2009) showed that upgrading mice home cages to an enriched environment not only reduced the reinforcing effect of psychostimulants, as it had been previously shown (Bardo et al., 2001; Bezard et al., 2003; El Rawas et al., 2009; Solinas et al., 2009), but completely eliminated cocaine-induced behavioral sensitization and CPP (Solinas et al., 2008). These results were later extended to additional drugs including methamphetamine, heroin and nicotine (Sikora et al., 2018).

Despite the fact that exposing animals to multiple rewards makes dissecting the neurobiological effects of each reward more complex, it brings preclinical models closer to the intricate human experience. Akin to Portugal, a few countries combat addiction and the social marginalization associated with it by offering treatment, support services and enforcing harm reduction policies (Cabral, 2017). These could be considered the clinical equivalent of enriched environment or social interaction, and the success of such drug policy supports the idea that, by implementing similar strategies, drug abuse can be greatly decreased.

## Models of Alcohol Intake

Similar to other drugs, alcohol abuse is a complex disorder impacted by social, economic and neurobiological factors (Goltseker et al., 2019). Aside from a few examples (Augier et al., 2014; de Guglielmo et al., 2017), voluntary alcohol consumption is typically weak, and often requires water-depriving the animals to incentivize drinking or initially pairing ethanol with a more salient reward, such as sucrose (Koob and Weiss, 1990; Becker, 2013; Goltseker et al., 2019). Since this “initiation training” introduces animals to multiple rewards and this can be problematic (see “Alternative Rewards” section above), a few rodent strains showing high preference for alcohol have been selectively bred (Li et al., 1979; Stewart and Li, 1997; Bell et al., 2006). Despite low levels of behavior, many studies use two-bottle choice and drinking in the dark (DID) models, both based on voluntary consumption. In the two-bottle choice model, animals are usually first presented with two bottles of water, later replacing one water solution bottle by another containing increasing percentages of alcohol (García Pardo et al., 2017). Access to alcohol can be continuous or intermittent, presenting the alcohol bottle only every other day (Brancato et al., 2016). Two-hours alcohol exposure is sufficient to measure significant correlations between alcohol intake and blood ethanol concentrations (Griffin, 2014). Several studies show that long-term exposure to intermittent alcohol access induces binge-drinking, potentiated alcohol preference and high blood alcohol concentrations (Wise, 1973; Carnicella et al., 2014). Because of these behaviors, this model mimics closely what is observed in humans, for whom the drinking pattern is a key factor in the development of alcohol use disorder (Kranzler and Soyka, 2018).

The DID model (Rhodes et al., 2005) takes advantage of rodent’s nocturnal activity to replace the home cage water bottle by a bottle containing a high concentration (20%) ethanol solution for a short period of time (2–4 h). This ethanol exposure promotes binge drinking and pharmacologically relevant blood ethanol concentrations, high enough to cause behavioral evidence of intoxication (Thiele and Navarro, 2014). This model aims to mimic the rapid and massive consumption most often observed in adolescent alcohol drinking. It does not require any modification of the alcohol solutions with other rewards or progressive increase of alcohol percentages, and binge drinking can be observed in 4 days, making it a simple and prompt model to use. However, the model seems to be somewhat restricted by mouse strain specificity. In the original study presenting the model (Rhodes et al., 2005), the DID paradigm induced binge drinking in the high ethanol drinking strain (C57BL/6J), yet the behavior was not observed in any of the other 11 inbred mice strains tested.

Chronic intermittent ethanol (CIE) is another recent model of alcohol use disorder gaining popularity (Griffin, 2014). This paradigm combines voluntary drinking and repeated exposure to alcohol vapor. After a 4-week training period of daily 2 h voluntary alcohol drinking, mice enter a cycle of 16 h vapor exposure, followed by 8 h of control air exposure (Lopez and Becker, 2005). After repeating the cycle 4–5 times (one cycle is enough to measure significant effects, but repeated cycles potentiate the behavioral outcome), animals are then tested in limited access sessions, similar to the ones performed during the training phase. CIE-animals exhibit a significant increase in voluntary ethanol drinking compared to controls, thus modeling the increase experienced by humans developing alcohol use disorder, that have been shown to be driven by neuroadaptations in glutamatergic and CRF signaling (Griffin, 2014). Since low voluntary alcohol consumption in most rodent strains is a notable limitation to study binge drinking (see discussion above), a non-contingent version of the CIE model, the Chronic-Intermittent Ethanol Administration (CIEA) paradigm, has been developed in rats (Nogales et al., 2014; Contreras et al., 2019) and mice (Sanchez-Roige et al., 2014; Lacaille et al., 2015; Monleón et al., 2019). The protocol follows the CIE timeline or a variation of it, i.e., repeated cycles of exposure to i.p. ethanol injections (3–4 g/kg) for several consecutive days intertwined with repeated days of non-exposure. CIEA is easy and inexpensive, and in combination with simple behavioral paradigms such as locomotion or elevated plus-maze, allows studying the neurobiology of binge drinking.

Alternative models focus on ethanol seeking behavior as a way to replicate relapse. Similar to other drugs, seeking behavior can be induced by priming injections of ethanol, stress, ethanol-paired cues or a combination of these factors (Le et al., 1998; Liu and Weiss, 2002; LeCocq et al., 2018). Using an alcohol-preferring rat strain, Giuliano et al. (2015) developed a different model of cue-induced alcohol-seeking. The procedure begins with a long exposure (18 sessions) to a 2-bottle choice procedure, followed by training to instrumental response to access alcohol paired with an alcohol-associated conditioned stimuli (CS). Alcohol seeking is measured during 20 min cycles where the drug is no longer present and contingent presentations of the CS act as a reinforcer. At the end of the seeking period, ethanol is re-introduced to avoid the CS losing its reinforcing properties. This experimental design aims to model alcohol craving by creating unusually high levels of alcohol-seeking behavior induced by the CS, followed by high alcohol consumption following the craving, thus closely mimicking craving leading to ethanol consumption in humans. Other models focus on compulsive-like alcohol intake, by incorporating aversive consequences to consumption, such as pairing alcohol intake with bitter quinine or footshocks (Hopf and Lesscher, 2014). These paradigms aim to model drug use despite negative consequences, one of the key symptoms listed in the DSM-5 to characterize substance abuse (APA, 2013). Quinine-resistant alcohol consumption seems to require long periods of ethanol exposure to develop, since it is measured after long cycles (8 months) of free access to alcohol (Spanagel et al., 1996; Fachin-Scheit et al., 2006; for review see Hopf and Lesscher, 2014) or after at least 3 months of intermittent access (Hopf et al., 2010). The models pairing footshocks to ethanol intake in alcohol-preferring rats show that the punishment context-dependently decreases subsequent alcohol SA (Marchant et al., 2013), however, some rats show footshock-resistant alcohol intake (Seif et al., 2013).

## Individual Variation in Addiction-Related Behaviors

It is widely acknowledged that regardless of the class of addictive drug, a minority of people who use the drug develop compulsive drug-seeking behaviors indicative of substance use disorder. Appropriately modeling this variation in animals can yield powerful insight into the neurobiological mechanisms that mediate addiction propensity. Fortunately, animal models exist that capture the individual variation inherently present in the human population. Impulsivity, a trait associated with addiction liability, can be assessed using an array of behavioral paradigms. Individual variation in the acquisition of drug-taking behavior can be captured using the high-responder (HR)/low-responder (LR) model, while individual variation in relapse propensity can be assessed using the sign-tracker (ST)/goal-tracker (GT) model. The HR/LR and ST/GT models are particularly useful as they capture individual variation in two distinct phases of drug addiction: acquisition and relapse, respectively.

### Models of Impulsivity

Although not specific to addiction, impulsivity, or the tendency to act prematurely without foresight, is often impaired in individuals with substance abuse disorders (Dalley et al., 2011; Ersche et al., 2012; Kaiser et al., 2016) and constitutes one of the risk factors for addictive behaviors (Dalley et al., 2007; Voon and Dalley, 2016; Kozak et al., 2019). This is particularly the case in adolescence and young adulthood, a critical period of substance experimentation, brain development and elevated impulsive behavior (Rømer Thomsen et al., 2018). Similarly, in preclinical models, adolescent animals (largely defined as postnatal day 21–60), tend to display more impulsive patterns of responding compared to adults (Burton and Fletcher, 2012; Hunt et al., 2016).

Adapted from a human task, the 5-choice serial reaction time task (5-CSRTT) rodent model was originally presented by Carli et al. (1983) in rats and has since become one of the most commonly used models to study attentional performance and motor impulsivity in rodents (Higgins and Silenieks, 2017). Rodents are first trained (20–30 sessions, around 100 trials/session) to respond to a visual stimulus on top of one of the five nose-pokes arranged on one wall of the testing chamber. Responding to the stimulus by poking the associated nose-poke is rewarded by food or liquid reward. This task requires the animal to maintain attention to the five nose-pokes and their corresponding visual cues. Poking during inter-trial intervals, i.e., between the last nose-poke and the presentation of a new stimulus, is recorded as a premature and impulsive response that is not rewarded and followed by a time-out. One of the advantages of this test resides in the possibility to control nearly every parameter, from the randomization of the visual stimuli to the length of the limited hold of the nose-poke and time out periods (Higgins and Silenieks, 2017). However, interpretation of the data requires researchers to take into account the extensive influence of attention when drawing conclusions, as any disorder disrupting attention might heavily alter the results. In this regard, using mice in this task could appear more delicate than using rats, since mice have shorter attention spans (Kentros et al., 2004; Hok et al., 2016). However, mice performances in this task are equivalent to rats, a few studies showing indeed superior motor control of task performance (Humby et al., 1999; Sanchez-Roige et al., 2012; Cope et al., 2016; Higgins and Silenieks, 2017). Exposure to drugs of abuse is typically correlated to robust increases in impulsive behavior, often specifically in adolescent rodents but not in adults, measured with the 5-CSRTT or the two-choice reaction time task, a simplified version of the former (Burton and Fletcher, 2012; Siemian et al., 2017; Moazen et al., 2018; Xue et al., 2018). Interestingly, a study compared cocaine and a cocaine-associated cue to compete for attention, and concluded that while cocaine severely disrupted the well-learned sustained attention task in rats, the cocaine-associated cue induced cocaine seeking but failed to impair the task (Pitchers et al., 2017c).

Another model focuses on response inhibition, i.e., the ability to inhibit a pre-potent (planned or already initiated) action by measuring action restraint and/or cancellation (Bari and Robbins, 2013). The animal is required to withhold responding for a set duration in order to receive a reward. Any premature response during the waiting interval resets the waiting time and increases the delay to reward.

Using these models, the field has established that impairments of the PFC, that undergoes profound pruning during adolescence (Drzewiecki et al., 2016), are both risk factors and consequences of impulse-control disorders, akin to what is observed in substance abuse (Goldstein and Volkow, 2011). Other studies show a role of VTA dopamine, locus coeruleus norepinephrine neurons and cholinergic neurotransmission on attention, impulsive and motivational control (Balachandran et al., 2018; Fitzpatrick et al., 2019; Sarter and Lustig, 2019).

### High-Responder/Low-Responder Model

Rats are characterized as HRs or LRs based on their cumulative locomotor movements during a locomotor test in a novel, inescapable environment, with HRs showing greater locomotor activity compared to LRs. This separates rats based on novelty-induced “sensation-seeking” behavior, a trait associated with drug addiction (Piazza et al., 1989; Dellu et al., 1996). This model captures individual variation in the acquisition of drug-taking behavior, specifically psychostimulants. Relative to LRs, HRs acquire cocaine (Piazza et al., 2000; Mantsch et al., 2001; Ferris et al., 2013), amphetamine (Piazza et al., 1989, 1990, 1991, 1998; Klebaur et al., 2001; Cain et al., 2008) and nicotine SA (Suto et al., 2001) at a faster rate. HRs also show greater behavioral sensitization to repeated amphetamine injections compared to LRs (Hooks et al., 1992). Despite acquiring drug-taking at different rates, outbred HRs and LRs do not differ in other addiction-related behaviors following prolonged cocaine SA, including the motivation to work for the drug, or drug-seeking behavior during tests of cocaine-primed and cue-induced reinstatement (Deroche-Gamonet et al., 2004). However, work using a model of rats selectively bred based on locomotor response to a novel environment [bred high-responder (bHR)/bred low-responder (bLR) model] has challenged this view, as these two phenotypes do differ in several addiction-related traits. Compared to bLR, bHR show higher levels of impulsivity (Flagel et al., 2010), attribute incentive motivational value to food and cocaine cues (Flagel et al., 2010), and acquire cocaine SA at a faster rate (Davis et al., 2008; Flagel et al., 2016). Recent work has shown that after prolonged SA training bHR initially acquires cocaine SA at a faster rate and show greater compulsive drug-seeking behavior when drug is not available compared to bLRs (Flagel et al., 2016). bHRs also show greater drug-seeking behavior during tests of cocaine-primed and cue-induced reinstatement compared to bLRs (Flagel et al., 2016). While the data from the selectively bred rat line (Flagel et al., 2016) contrasts those of an outbred population of rats (Deroche-Gamonet et al., 2004), it appears that this model may still be relevant for assessing individual variation in addiction-related behavior beyond the acquisition of drug-taking.

Work focusing on the neurobiological mechanisms underlying phenotypic differences between HRs and LRs has focused mainly on the mesolimbic dopamine system. Following drug experience, both outbred and selectively bred HRs and LRs differ in several dopamine parameters within the NAc (Rougé-Pont et al., 1993; Chefer et al., 2003; Flagel et al., 2010; Ferris et al., 2013; Waselus et al., 2013; Mabrouk et al., 2018), as well as dopamine firing rates in the VTA (McCutcheon et al., 2009, HRs and LRs only). Differences also exist in basal levels of epigenetic modification within the NAc in bHRs and bLRs (Chaudhury et al., 2014), and these differences persist following cocaine experience (Flagel et al., 2016). In addition to the mesolimbic dopamine system, HRs and LR differentially engage the hypothalamic-pituitary-adrenal axis (Piazza et al., 1991; Kabbaj et al., 2007), which is also believed to contribute to differences in addiction-related behaviors between the two phenotypes.

### Sign-Tracker/Goal-Tracker Model

The sign-tracker (ST)/goal-tracker (GT) model is used to assess individual variation in the motivational value of a reward-paired cue during a Pavlovian conditioned approach (PavCA) task (Flagel et al., 2007; Robinson and Flagel, 2009; Meyer et al., 2012). During Pavlovian learning, a once neutral stimulus that reliably precedes the delivery of a reward becomes attributed with a predictive value and is transformed into a CS (Pavlov, 1927). However, in addition to a predictive value, the CS can also be attributed with an incentive motivational value and invigorate behavior on its own (Robinson and Berridge, 1993; Berridge, 2001). During PavCA training, rats that attribute a predictive value to the CS are called GTs, whereas those that attribute both a predictive and incentive motivational value to the CS are STs. Using this model, the neurobiological mechanisms underlying the predictive vs. the incentive motivational value of a reward cue have been explored. STs engage regions within the “motive circuit” (Kalivas and Volkow, 2005) to a greater extent than GTs in response to both food (Flagel et al., 2011a; Haight et al., 2017) and drug-paired cues (Yager et al., 2015). Dopamine transmission within the NAc core (Flagel et al., 2007, 2011b; Saunders and Robinson, 2012) and PFC (Pitchers et al., 2017b) also mediates sign-tracking behavior, whereas cholinergic transmission within the PFC mediates goal-tracking behavior (Pitchers et al., 2017b). Collectively, work has shown that goal-tracking behavior is reliant on “top-down” cortical processing (for review see Kuhn et al., 2018a; Sarter and Phillips, 2018; Campus et al., 2019), whereas sign-tracking behavior engages “bottom-up” subcortical processing (for review see Flagel and Robinson, 2017; Kuhn et al., 2018a). It is proposed that the imbalance between “top-down” and “bottom-up” processing results in the behavioral differences between the phenotypes.

It has been postulated that attributing an excessive incentive motivational value to a reward-cue can lead to maladaptive behaviors such as drug addiction. In fact, STs and GTs differ in several addiction-related behaviors. For example, in addition to sign-tracking to cues associated with a food reward, STs also sign-track to cues associated with cocaine (Uslaner et al., 2006; Yager and Robinson, 2013) and opioid (Yager et al., 2015) reward delivery. Relative to GTs, STs are also more impulsive (Flagel et al., 2010; Lovic et al., 2011) and will work harder for an infusion of cocaine (Saunders and Robinson, 2011). However, the two phenotypes do not differ in the rate of cocaine SA (Saunders and Robinson, 2010; but see Beckmann et al., 2011; Saunders et al., 2013; Kawa et al., 2016; Kuhn et al., 2018b) or operant extinction (Saunders and Robinson, 2011; Kawa et al., 2016; Kuhn et al., 2018b), but do differ in reinstatement of drug-seeking behavior. STs show greater rates of both cocaine-primed (Saunders and Robinson, 2011) and cue-induced (Saunders et al., 2013; but see Kawa et al., 2016) reinstatement of drug-seeking behavior compared to GTs following ShA training. Work has shown that enhanced dopamine transmission within the NAc core contributes to higher cue-induced drug-seeking behavior observed in STs (Saunders et al., 2013). Additionally, the paraventricular nucleus of the thalamus, a region within the motive circuitry (Kelley et al., 2005) that has recently gained attention for mediating motivated behaviors including addiction-related behaviors (for review see Millan et al., 2017), is also a key node regulating this individual variation in cue-induced drug-seeking behavior (Kuhn et al., 2018b).

Though STs are more susceptible to both cocaine-primed and cue-induced reinstatement, GTs show greater drug-seeking behavior during a test for context-induced reinstatement (Saunders et al., 2014), and in response to discriminative stimuli associated with reward delivery, an effect mediated by cholinergic transmission within the PFC (Pitchers et al., 2017a). Compared to STs, GTs more readily utilize cortical processing (for review see Sarter and Phillips, 2018), and it is postulated that this cortical engagement allows them to disentangle the complex nature of contexts and discriminative stimuli better than STs, resulting in more drug-seeking behavior. This difference in relapse propensity between STs and GTs relative to the type of reinstatement implies that both phenotypes are sensitive to addiction-related behaviors; however, the environmental contingencies and neurobiological mechanisms mediating these effects differ. These findings suggest that in contrast to the HR/LR model, the main strength of the ST/GT model is elucidating the neurobiological mechanisms associated with individual variation in relapse propensity. This model also has translational validity, as work in humans has shown sign- and goal-tracking behavior (Garofalo and di Pellegrino, 2015; Joyner et al., 2018; Schad et al., 2019), though linking a specific conditioned response in humans with addiction-related behaviors has yet to be explored.

## Additional Models of Behavioral Addictions

### Gambling Disorder

Due to its diagnostic similarities with substance-use disorders, gambling disorder (GD; Langdon et al., 2019) was moved to a new category entitled “Substance-related and Addictive Disorders” in the most recent DSM (APA, 2013). In fact, GD is currently the only behavioral addiction that is diagnosable in the DSM, and interestingly there is a high level of comorbidity between individuals diagnosed with GD and drug addiction (Lorains et al., 2011). GD is characterized as compulsive gambling behavior that results in distress and causes disruptions to an individual’s personal and professional life (APA, 2013). The Iowa Gambling Task (IGT), a neuropsychological battery used to assess decision-making strategies, is clinically used to study GD. During this task, an individual selects a card from one of four decks, each of which has a different probability of a reward to punishment ratio assigned to it resulting in “safe” and “risky” options (Bechara et al., 1994). Individuals with GD choose cards from riskier decks more often than healthy control, resulting in fewer winnings (Cavedini et al., 2002). In fact, performance on this task has been associated with treatment success (Alvarez-Moya et al., 2011).

The rodent gambling task (rGT; Zeeb et al., 2009) is the most commonly used adaptation of the IGT. During this task, rats have the option to poke into four different ports, and each port has a specific reward (e.g., sugar pellets) and punishment (e.g., time-out period between trials) ratio associated with it. Rats are given a fixed amount of time to complete the task and choosing from the riskier ports (i.e., more sugar pellets but a longer time-out period) results in a lower net gain compared to selecting the safer ports. Studies have found that the orbitofrontal cortex (OFC) and the basolateral amygdala (BLA) mediate the acquisition of adaptive decision making during this task (Zeeb and Winstanley, 2011, 2013). However, only the BLA is needed for the continued expression of the adaptive strategy suggesting that the OFC is initially recruited to establish the behavior, but the BLA maintains it (Zeeb and Winstanley, 2011; Zeeb et al., 2015). The insular cortex has also been shown to mediate decision making during the rGT (Ishii et al., 2015; Pushparaj et al., 2015; Daniel et al., 2017). These data complement studies in humans demonstrating that various regions of the PFC are recruited during the IGT (Fellows and Farah, 2005; Tanabe et al., 2007; Lawrence et al., 2009; Power et al., 2012), and the amygdala mediates GD in humans (Bechara et al., 1999; Takeuchi et al., 2019).

While the rGT emulates aspects of GD and appears to have translational significance, it has been criticized for only modeling poor decision-making strategies, and not behaviors exclusively associated with GD (for review see Winstanley and Clark, 2016). To better address this, additional rodent models have been developed to model specific aspects of GD. For example, the rodent betting task models “escalation of commitment”, or rather the phenomenon that people become more cautious as the stakes get higher (Staw, 1981). Like humans, as stakes get higher rats will more often select a certain reward as opposed to the chance of receiving a higher reward with the risk of receiving nothing at all (Cocker et al., 2012). The BLA (Tremblay et al., 2014), regions within the PFC (Barrus et al., 2017), and dorsal striatal dopamine levels (Cocker et al., 2012) appear to mediate behavior during this task. Gambling tasks have also been created to mimic the behavior of “loss-chasing,” or continuing to gamble in an effort to earn back previous losses, which is commonly observed in individuals with GD (Toce-Gerstein et al., 2003; Strong and Kahler, 2007). During this paradigm, rats intermittently must choose between withholding responding during a time-out period, or to gamble with the chance of avoiding the time-out period with the risk of doubling it (Rogers et al., 2013). The BLA (Tremblay et al., 2014), as well as serotonin and dopamine transmission (Rogers et al., 2013), have been shown to mediate behavior during this task. Lastly, a task known as the rat slot machine task has been used to model the “near-miss effect” (Peters et al., 2010) seen in humans whereby barely losing a gamble motivates continual gambling (Kassinove and Schare, 2001; Clark et al., 2009). During this task, rats must first poke into all available ports, after which the lights within each port either turn on or stay off. If all lights come on, that is considered a “win” trial and the rat must press on a lever in order to receive the reward. Compared to other “loss” trials, rats show greater lever pressing when a “near miss” occurs (e.g., lights in all ports come on except one vs. only one light coming on; Winstanley et al., 2011). Dopamine signaling has been implicated in mediating behavior during this task (Winstanley et al., 2011; Cocker et al., 2014, 2017), particularly within the insular cortex (Cocker et al., 2016).

Other factors that affect gambling behavior in humans are also being taken into consideration when modeling GD, such as the motivational salience of audiovisual cues that are commonly present in casinos. Similar to behavior in drug addiction, cues and contexts associated with gambling have been shown to affect gambling behavior (for review see Barrus et al., 2016), and behavioral paradigms in rodents have been created to emulate these effects (Barrus and Winstanley, 2016; Adams et al., 2017; Langdon et al., 2019). Taken together, it is evident that no single behavioral paradigm can capture the complexity of GD in rodents. Thus, similar to studying drug addiction-related behaviors in animal models, a battery of assessments are used in order to better model and assess the neurobiological underpinnings of GD.

### Exercise Addiction

Though not included in the DSM, exercise addiction (EA) has been reported to affect 0.3–0.5% of the general population and 1.9–3.2% of individuals who regularly exercise (Berczik et al., 2012; Griffiths et al., 2015). The main issue in diagnosing individuals with EA is that diagnostic criteria have not yet been agreed upon. Several screening tools have been developed to assess EA in humans, including the Exercise Addiction Inventory and the more commonly used Exercise Dependence Scale (for review see Hausenblas et al., 2017). Several studies have proposed that criteria for EA be adapted from those used to diagnose substance use disorders per the DSM, including measures of tolerance, withdrawal, and decreased involvement in other activities (for review see Freimuth et al., 2011). Independent of diagnostic criteria, studies have identified behavioral traits associated with EA, such as obsessive-compulsive behavior, loneliness and anxiety (Macfarlane et al., 2016; Lukács et al., 2019). Several other disorders, including substance use disorders, eating disorder, and other behavior addictions such as shopping and sex addiction have been found to co-occur with EA (for review see Freimuth et al., 2011). Despite not having a standardized diagnostic procedure, rodent models of EA exist, primarily composed of assessing wheel-running behavior. In fact, several studies using rodent wheel-running models have yielded evidence of behaviors akin to symptoms of EA aligning with those observed in substance use disorders (for review see Richter et al., 2014). Though still being fully conceptualized, it is evident that EA is garnering more attention and is being more rigorously assessed in both clinical and preclinical models.

## Models Used by Pharmaceutical Companies to Assess Abuse Potential and Their Face Validity

Regardless of the scientific question, all animal models strive to represent part of the neurobiological mechanisms that guide human behavior. Models are limited in this regard, but their use is needed to assess the abuse potential of new drugs. We describe in this section a general overview of the procedures commonly used by pharmaceutical companies during a new drug safety evaluation to assess that risk. In 2017, the Food and Drug Administration agency (FDA) released an updated guide for industry gathering nonbinding recommendations on how to evaluate whether a new drug product acting on the central nervous system (CNS) has abuse potential and required to be subject to control under the Controlled Substances Act (CSA; U.S. Department of Health and Human Services, 2017). FDA does not recommend that every drug under development undergo an evaluation of abuse potential, but proposes focusing on the new CNS-active molecular entities that have not previously been assessed by FDA for abuse potential. After assessing if a new drug, or any of its major metabolites, is CNS-active through chemistry, pharmacokinetics and receptor-binding studies, the next step includes abuse-related animal behavioral studies, most commonly performed in rats. A first set of safety studies measure the effects of the drug on general behavior, such as a motor performance test. As an example, drug-induced hyperactivity is recorded as an abuse-related signal. Specific abuse-related studies evaluate: (1) the rewarding and reinforcing properties of the drug; and (2) the similarity of the effects of the new drug to established drugs of abuse, defined as drug discrimination. The rewarding and reinforcing properties are measured using SA starting on fixed ratio 1, which increases with continual training. Drug discrimination generally involves training animals to self-administer a training drug, usually a drug of abuse of similar classification than the newly tested drug, with a known mechanism of action. When the new drug induces the same SA behavior than the training drug, it is then hypothesized that drugs share a pharmacological mechanism of action (Carter and Griffiths, 2009). However, since certain classes of drugs, such as hallucinogenic 5HT_2A_ agonists or cannabinoids, are poorly self-administered if at all (Yanagita, 1986; Fantegrossi et al., 2004; Heal et al., 2018), conditioned place-preference studies are alternatively recommended to establish potential rewarding properties of a new drug (Heal et al., 2018). These studies are usually performed at the end of phase 2 of the clinical trial, using the final therapeutic doses previously selected. When the studies establishing drug reward properties are completed, the FDA additionally recommends performing a physical dependence study, to identify potential withdrawal syndromes and behavioral disruptions upon abrupt drug discontinuation.

The decision to test the abuse potential of a new drug in humans depends on the results of the above-mentioned animal studies, a thorough comparison of the preclinical and clinical studies related to the drug and profiles of abuse- and euphoria-related adverse events established in healthy individuals and individuals with the disease of study. If all of these markers point to abuse-related signals, the drug is then tested in humans reporting numerous recent recreational experiences with drugs in the same drug class as the tested drug, through human abuse potential additional studies.

The two major models used to classify and regulate drug use, SA and CPP, present different levels of face validity. SA, including multiple-choice SA procedures (Griffiths et al., 1993), presents a high level of face validity and predictive validity (Haney and Spealman, 2008; Carter and Griffiths, 2009) and drugs self-administered in rodents correspond with those having reinforcing properties in humans (Schuster and Thompson, 1969; Griffiths and Ator, 1980; Haney and Spealman, 2008; Carter and Griffiths, 2009). In several studies, preclinical identification of drugs reducing craving and/or intake successfully translated to humans (Heilig et al., 2016). This was the case for the α2-adrenergic agonist clonidine that reduced cocaine and heroin craving in a human laboratory setting and clinical trial (Jobes et al., 2011; Kowalczyk et al., 2015). Similarly, glucocorticoid receptor antagonist mifepristone was found to decrease alcohol intake in rats, and also lessened cue-induced alcohol craving and intake in individuals with alcohol use disorder (Vendruscolo et al., 2012, 2015).

It is critical to remember that although useful, simple rodent models fail to encapsulate the complexity of human life. Factors such as drug availability, economic and socio-cultural levels influence the opportunities and experiences available to individuals and thus impact the risk of abuse. Preclinical models offering alternative rewards or including behavioral economics tackle some of these factors (see above), but these models are mostly limited to laboratory use currently and are not yet mainstreamed for pharmacological testing. Some authors additionally argue that, albeit the current models used to assess abuse potential are valid and reliable, they often lack accuracy assessing the propensity to develop addiction and the severity in which it will manifest, two parameters deeply influenced by individual vulnerability (Conway et al., 2010). They argue the need for better indices accounting for the complexity and multifactorial traits that impact the development of addiction in humans, in order to develop improved prevention approaches and treatment strategies.

One way to address these limitations is to introduce genetic variability in rodent models. While genetic factors contribute to susceptibility to drug addiction (for review see Nestler, 2000; Bevilacqua and Goldman, 2009), the heritability of drug use varies with hallucinogens showing the lowest level of heritability and cocaine showing the highest (Goldman et al., 2005). Rat lines exist that inherently capture genetic diversity, such as the heterogeneous stock rat, allowing for better representation of genetic and behavioral variation present in the human population (Solberg Woods, 2014; Woods and Mott, 2017). For a more targeted assessment of the role a gene may have in a behavioral trait, transgenic models, specifically genetic knockout and overexpressing models, can be used. In contrast to manipulating the genetic background of an animal to assess changes in behaviors, models can also be created by selectively breeding animals based on a specific behavioral trait, such as in the bHR/bLR model, and then assessing genetic differences between phenotypes. Lastly, to gain a broader understanding of the genetic differences that may contribute to certain phenotypic traits in animal models, quantitative trait loci analysis can be used. Using this technique, genetic variants can be identified in animals that differ in behaviors associated with addiction. While genetics plays a role in the predisposition for addiction-related behaviors, previous work has also focused on the epigenetic effects drugs of abuse have on the long-term transcriptional regulation of genes and how this contributes to addiction (Robison and Nestler, 2011). By focusing on the genetic basis for addiction-related behaviors, in conjunction with implementing better preclinical models of addiction, pharmaceutical companies can identify more successful therapeutics interventions in the treatment of drug addiction.

## Conclusion

Despite the complexity of substance-related and addictive disorders, we have highlighted in this review currently used preclinical models aiming to mimic as closely as possible the behaviors observed in humans. As models become more complex, the tools used to study the underlying neurobiological substrates also improve, moving the field forward towards future therapeutic opportunities.

## Author Contributions

All authors listed have made a substantial, direct and intellectual contribution to the work, and approved it for publication.

## Conflict of Interest

The authors declare that the research was conducted in the absence of any commercial or financial relationships that could be construed as a potential conflict of interest.

## Abbreviations

5-CSRTT: 5-Choice Serial Reaction Time Task
AMPA: Receptor: α-amino-3-hydroxy-5-methyl-4-isoxazolepropionic acid receptor
BLA: Basolateral amygdala
bHR: Bred high-responder
bLR: Bred low-responder
BP: Break-point
CIE: Chronic intermittent ethanol
CNS: Central nervous system
CPA: Conditioned Place Aversion
CPP: Conditioned place preference
CS: Conditioned stimulus
CSA: Controlled Substances Act
DID: Drinking In the Dark
DS: Discriminative stimulus
DSM: Diagnostic and Statistical Manual
EA: Exercise addiction
GD: Gambling disorder
GT: Goal-tracker
FDA: Food and Drug Administration agency
HR: High-responder
IGT: Iowa gambling task
IntA: Intermittent access training
LgA: Long-access training
LR: Low-responder
NAc: Nucleus accumbens
OFC: Orbitofrontal cortex
PavCA: Pavlovian conditioned approach training
PFC: Prefrontal cortex
rGT: Rodent gambling task
SA: Self-administration
ShA: Short-access training
ST: Sign-tracker
THC: Δ^9^-tetrahydrocannabinol
VTA: Ventral tegmental area.
